# Supplementary material for: Magmas Inhibition in Prostate Cancer: A Novel Target for Treatment-Resistant Disease
Source: Cancers (Basel). 2022 May 31;14(11):2732. doi: 10.3390/cancers14112732 (PMC9179500; doi:10.3390/cancers14112732)
Supplement: Supplementary file 1 [file cancers-14-02732-s001.zip › cancers-1690907-supplementary.pdf]

Supplementary Table S1: Description of datasets used from Oncomine/cBioPortal databases.

Database	Name of dataset	Description of samples	Accession Number	Reference Number
Oncomine	Arredouani Prostate	13 prostate cancer and 8 normal prostate samples	GSE55945	15
Oncomine	Grasso Prostate	59 localized prostate cancer and 35 metastatic castrate resistant prostate cancer with 28 matched benign prostate samples	GSE35988	16
Oncomine	Lapointe Prostate	62 prostate cancer and 41 normal prostate samples		17
Oncomine	Liu Prostate	44 prostate cancer and 13 normal prostate samples		18
Oncomine	Tamura Prostate	25 hormone-resistant prostate cancer and 10 hormone-sensitive prostate cancer with 5 normal prostate samples	GSE6811	19
Oncomine	Taylor Prostate	150 prostate cancer and 29 normal prostate samples	GSE21032	20
Oncomine	Vanaja Prostate	32 prostate cancer and 8 normal prostate samples		21
Oncomine	Varambally Prostate	5 localized prostate cancer and 4 metastatic prostate cancer with 4 benign prostate samples	GSE3325	22
cBioPortal	Neuroendocrine Prostate Cancer (Multi-Institute, Nat Med 2016)	114 metastatic prostate cancer tumor/normal pairs		24
cBioPortal	Prostate Adenocarcinoma	176 tumors from 63 patients with		25

	(Fred Hutchinson CRC, Nat Med 2016)	disseminated prostate cancer with matched normals.		
cBioPortal	Prostate Adenocarcinoma (MSK, Eur Urol 2020)	1465 prostate adenocarcinoma tumor/normal pairs		26

Contd.

Database	Name of dataset	Description of samples	Accession Number	Reference Number
cBioPortal	Metastatic Prostate Cancer (SU2C/PCF Dream Team, Cell 2015)	150 metastatic prostate cancer tumor/normal pairs		27
cBioPortal	Prostate Adenocarcinoma (TCGA, Cell 2015)	333 primary prostate adenocarcinoma tumor/normal pairs		23
cBioPortal	Prostate Adenocarcinoma (TCGA, PanCancer Atlas)	494 primary prostate adenocarcinoma tumor/normal pairs		
cBioPortal	Prostate Adenocarcinoma (TCGA, Firehose Legacy)	501 primary prostate adenocarcinoma tumor/normal pairs		

Supplementary Table S2: statistical analyses with P-values

Table S2-1. Data and *P* Value for Figure 4A

	Without Z-VAD	With Z-VAD	*P* Value
BT#9 (10µM)	51.2±4.8	54.7±2.3	**0.325**
BT#9 (20µM)	26.5±5.5	27.7±4.8	**0.774**

Table S2-2. Data and *P* Value for Figure 5E

NAC	BT#9 (10µM)	***p* Value**	BT#9 (20µM)	***p* Value**
0	47.5±6.2	-	28.6±2.3	-
10 µM	60.6±6.8	**0.068**	33.7±4.0	**0.131**
100 µM	70.5±6.2	**0.011**	44.8±6.6	**0.016**
1000 µM	78.1±3.9	**0.002**	62.4±6.2	**0.001**

Table S2-3. Data and *P* Value for Figure 6A

	**TRAIL Alone**	**+ BT#9**	***p* Value**
**TRAIL10ng/ml**	7.5±1.5	13.8±4.7	**0.045**
**TRAIL 100ng/ml**	17.5± 5.6	28.0±3.0	**0.022**

Table S2-4. Data and *P* Value for Figure 6C

	**Docetaxel alone**	**+ BT#9**	***p* Value**
**Docetaxel 0.1µM**	11.0±2.6	14.2±7.3	**0.516**
**Docetaxel 1µM**	23.2± 3.9	24.6±3.2	**0.669**

Supplementary Figure S1: densitometry intensity ratio and uncropped picture of Figure 3B

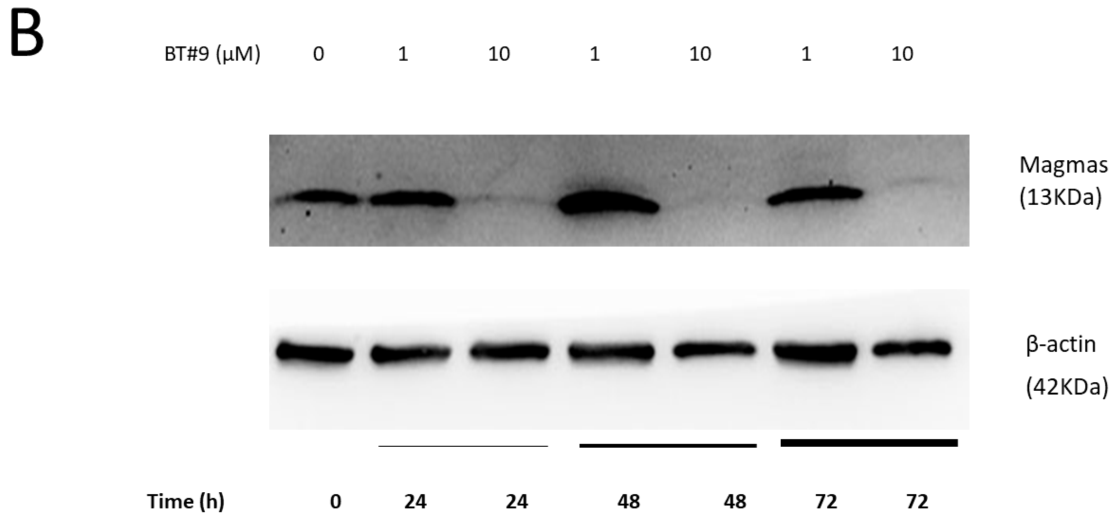

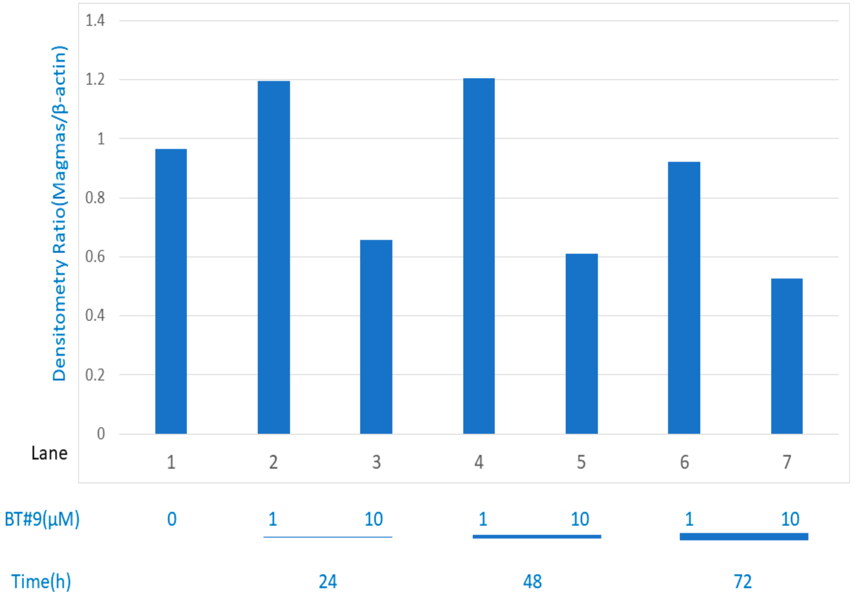
Densitometry Ratio(Magmas/β-actin)
1.4
1.2
1
0.8
0.6
0.4
0.2
0
Lane
1
2
3
4
5
6
7
BT#9(μM)
0
1
10
1
10
1
10
Time(h)
24
48
72

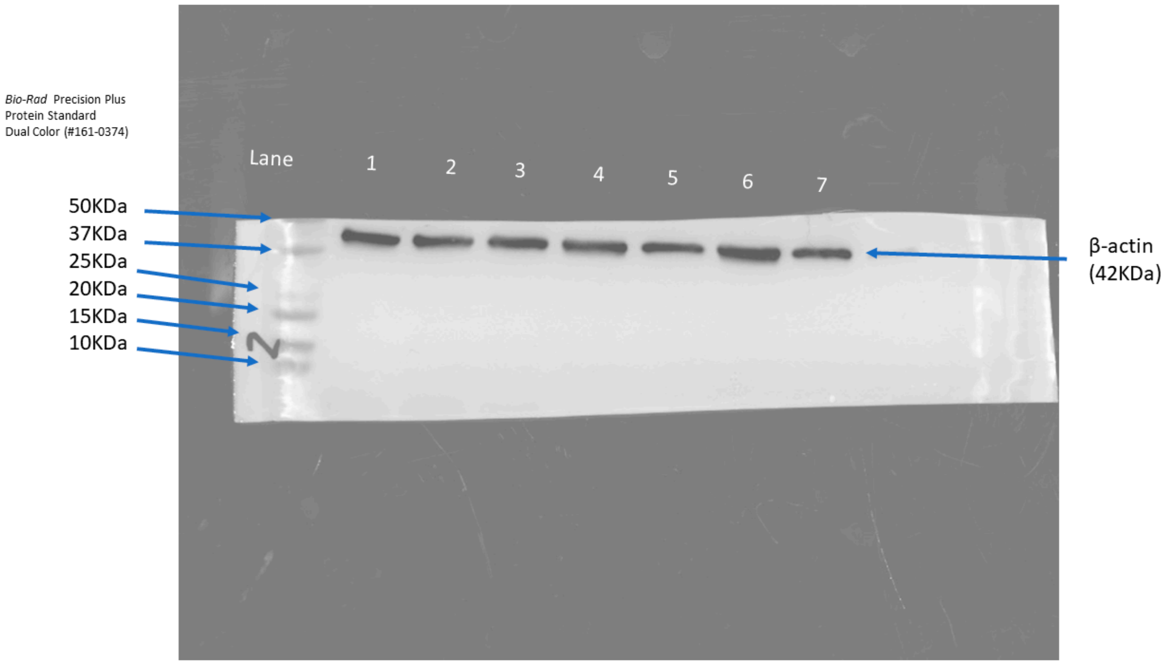
Bio-Rad Precision Plus
Protein Standard
Dual Color (#161-0374)
Lane
1
2
3
4
5
6
7
50KDa
37KDa
25KDa
20KDa
15KDa
10KDa
β-actin
(42KDa)

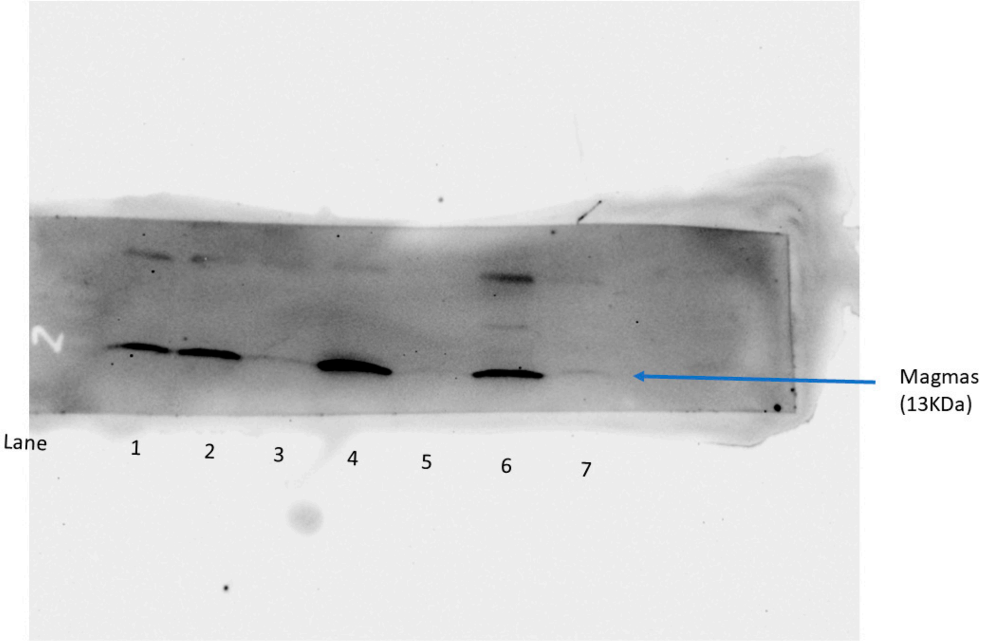

Magmas
(13KDa)
Lane
1
2
3
4
5
6
7

Supplementary Figure S2(Magnified Figure 4): The mode of cell death treated by BT#9)

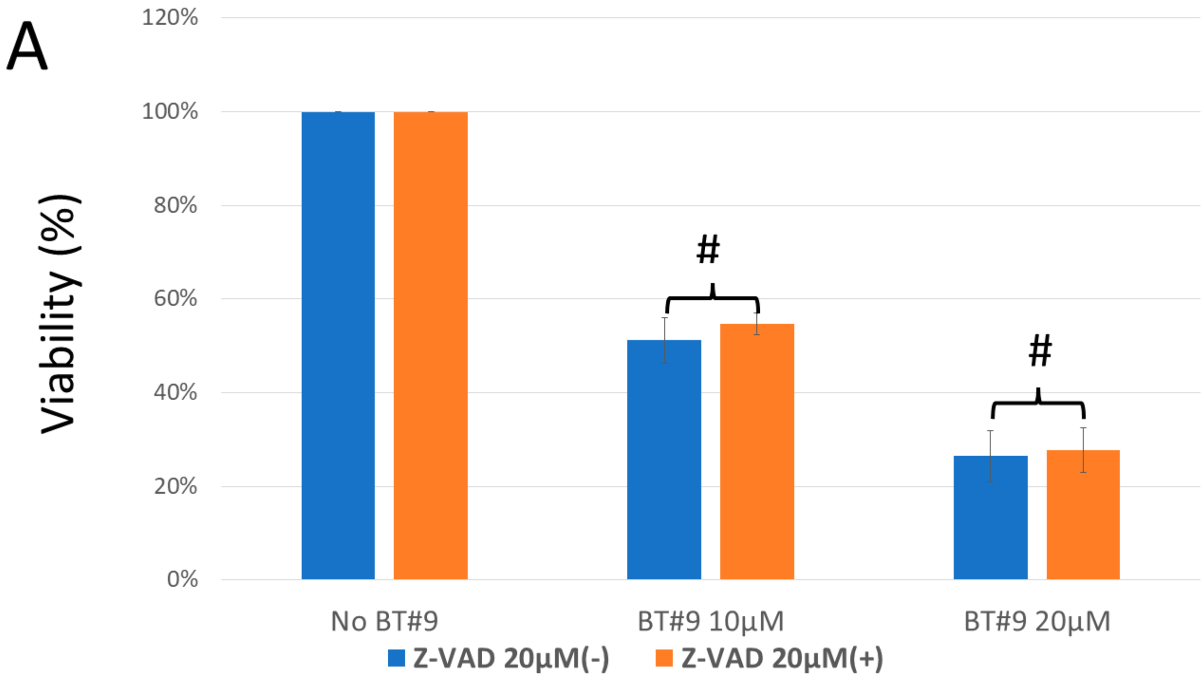

Supplemental Figure S2A (Magnified Figure 4A): MTS viability assay showed that General Caspase Inhibitor(Z-VAD) did not protect BT#9 induced cell death in PC-3 cells (#: P>0.05).

B

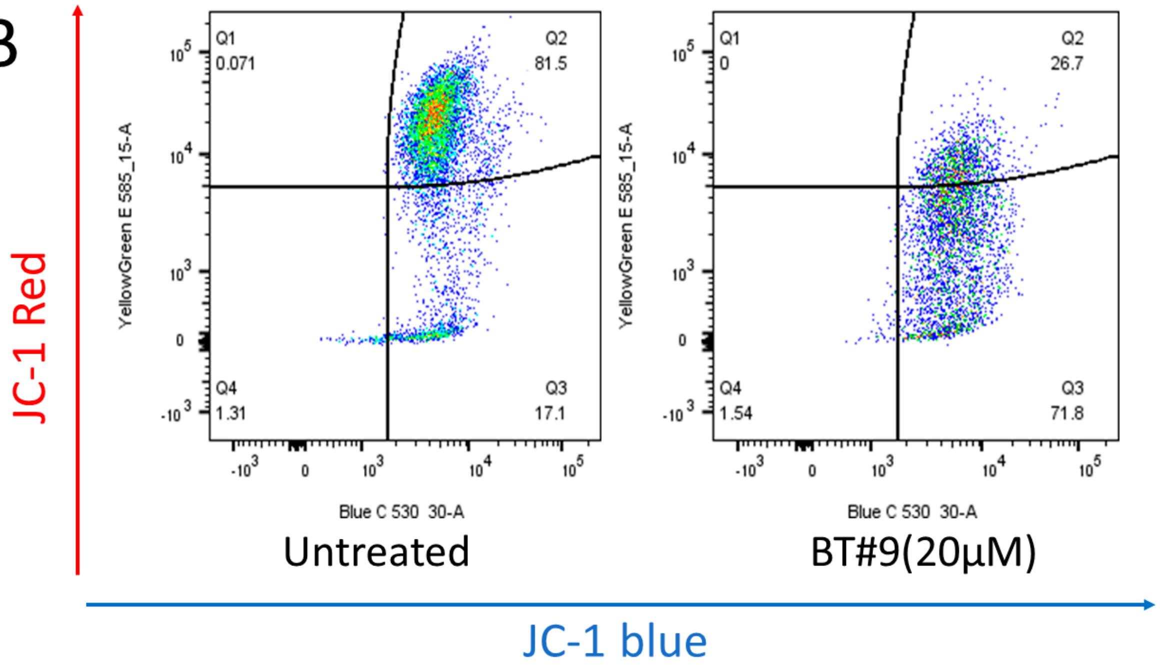

Supplemental Figure S2B (Magnified Figure 4B): Dot plot of fluorescence shift in PC-3 cells after treated with 20 μM BT#9 for 4 h. The ratios of red/green(Q2/Q3) were decreased from 81.5/17.3(untreated) to 26.7/71.8 (treated), indicating the loss of MMP.

C

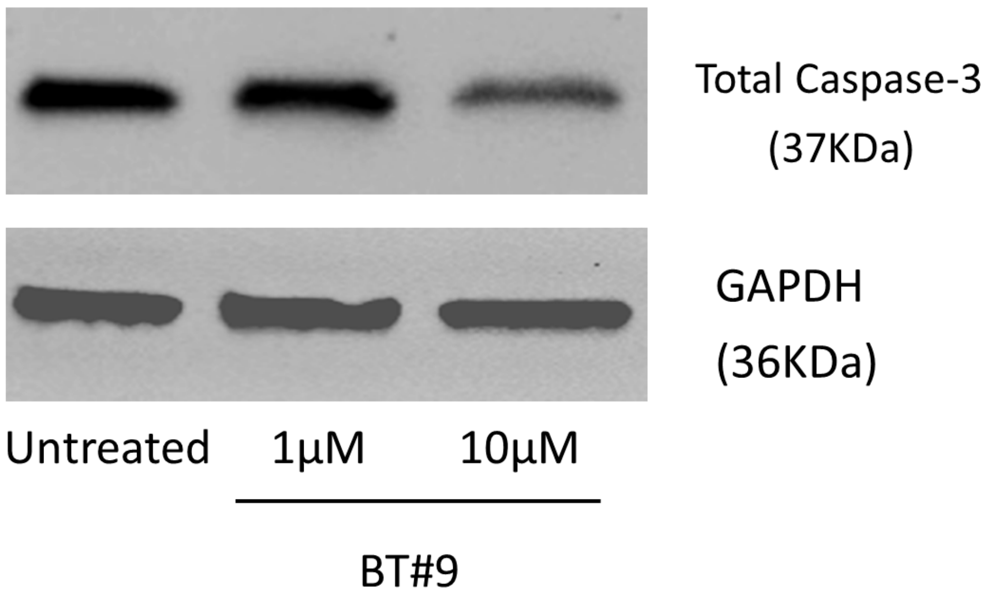

Total Caspase-3
(37KDa)
GAPDH
(36KDa)
Untreated
1μM
10μM
BT#9

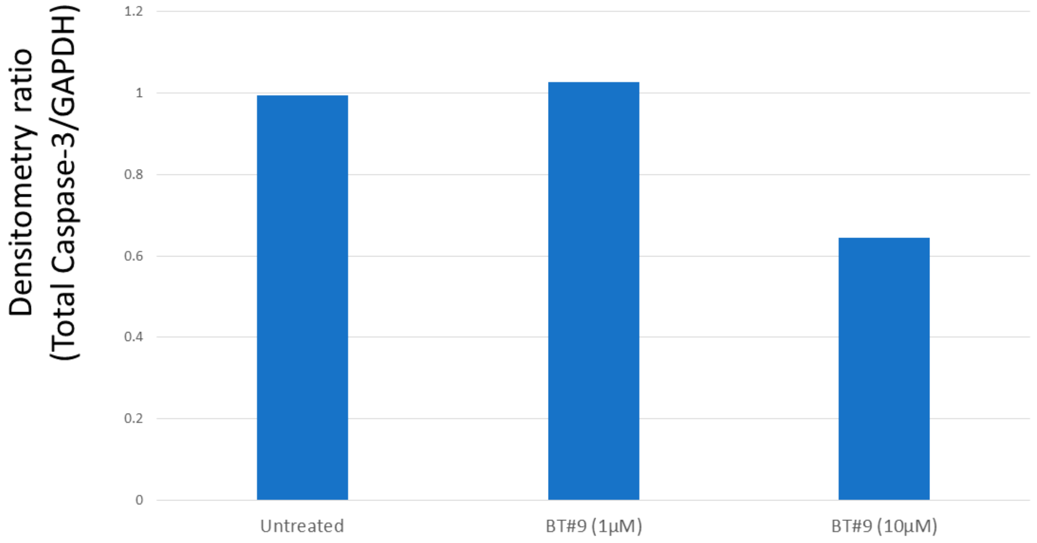

Densitometry ratio
(Total Caspase-3/GAPDH)
1.2
1
0.8
0.6
0.4
0.2
0
Untreated
BT#9 (1μM)
BT#9 (10μM)

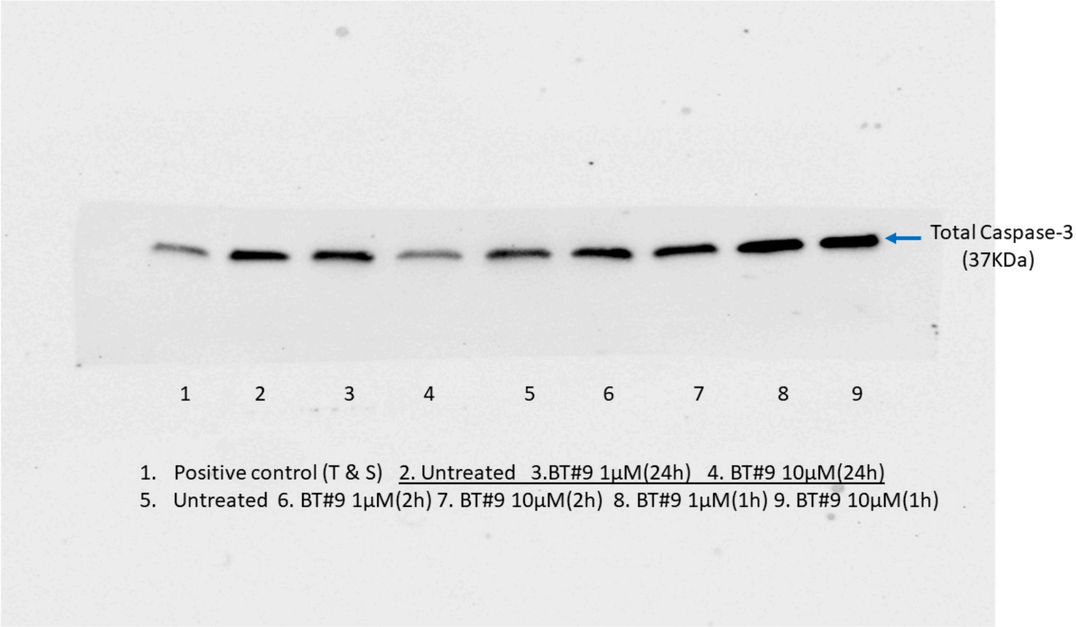

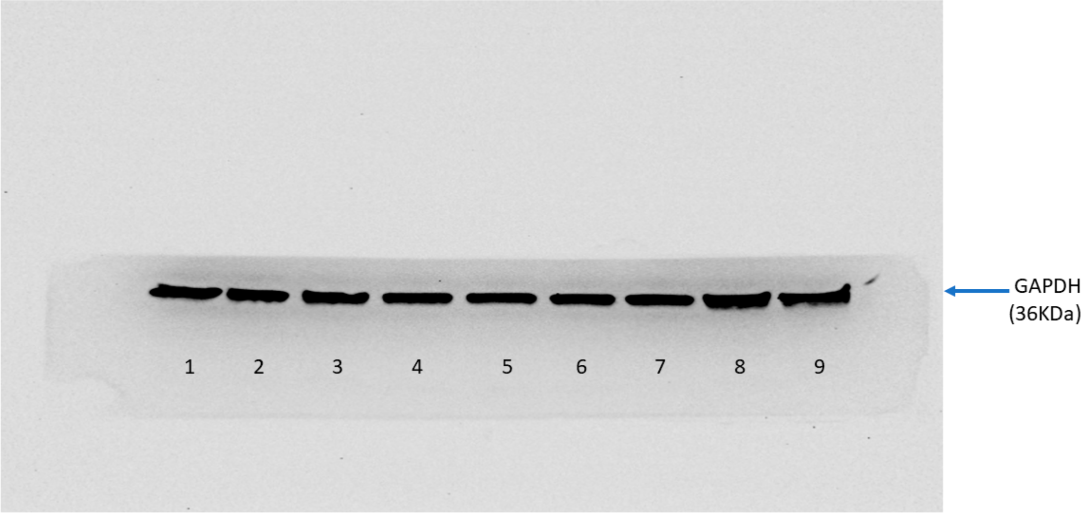

1. Positive control (T & S) 2. Untreated 3.BT#9 1µM(24h) 4. BT#9 10µM(24h)
5. Untreated 6. BT#9 1µM(2h) 7. BT#9 10µM(2h) 8. BT#9 1µM(1h) 9. BT#9 10µM(1h)

Supplemental Figure S2C (Magnified Figure 4C): PC-3 cells were treated by 1 µM or 10µM BT#9 for 24 hours, Western blot showed decreased total caspase-3, indicating the activation of caspase-3. Positive control (T & S, TRAIL 100ng/ml and SMAC mimic 1000nM).

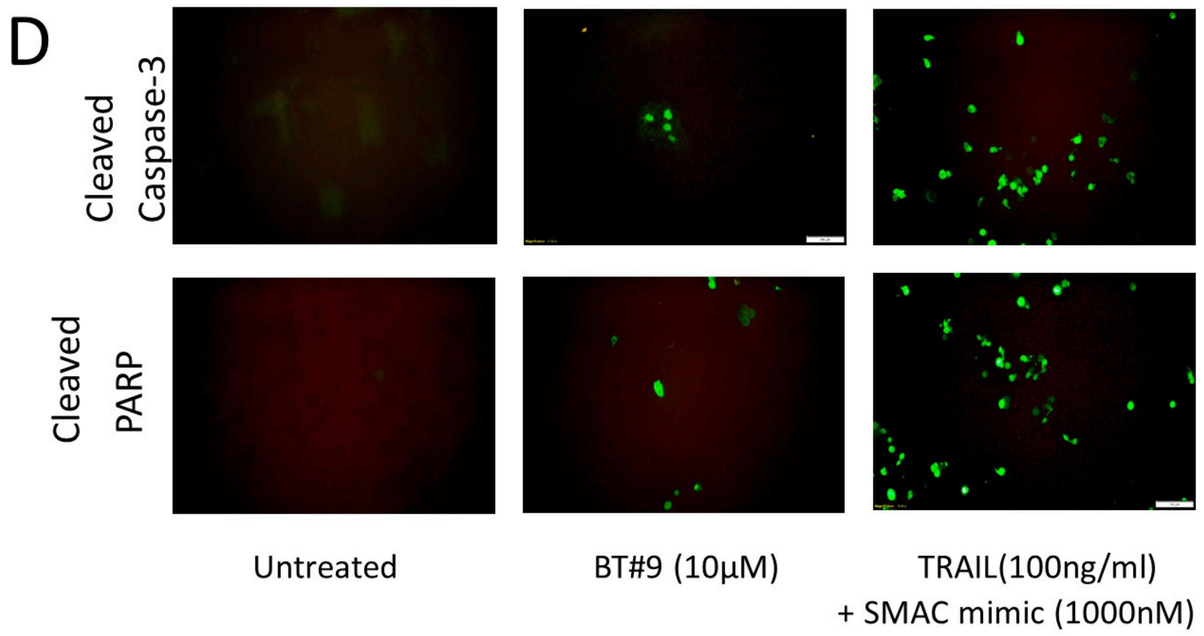

Supplemental Figure S2D (Magnified Figure 4D): Fluorescence microscopy also confirmed that smaller percentage of cleaved caspase-3 and cleaved PARP in PC-3 cells treated by 10 μM BT#9 when compared with positive control.

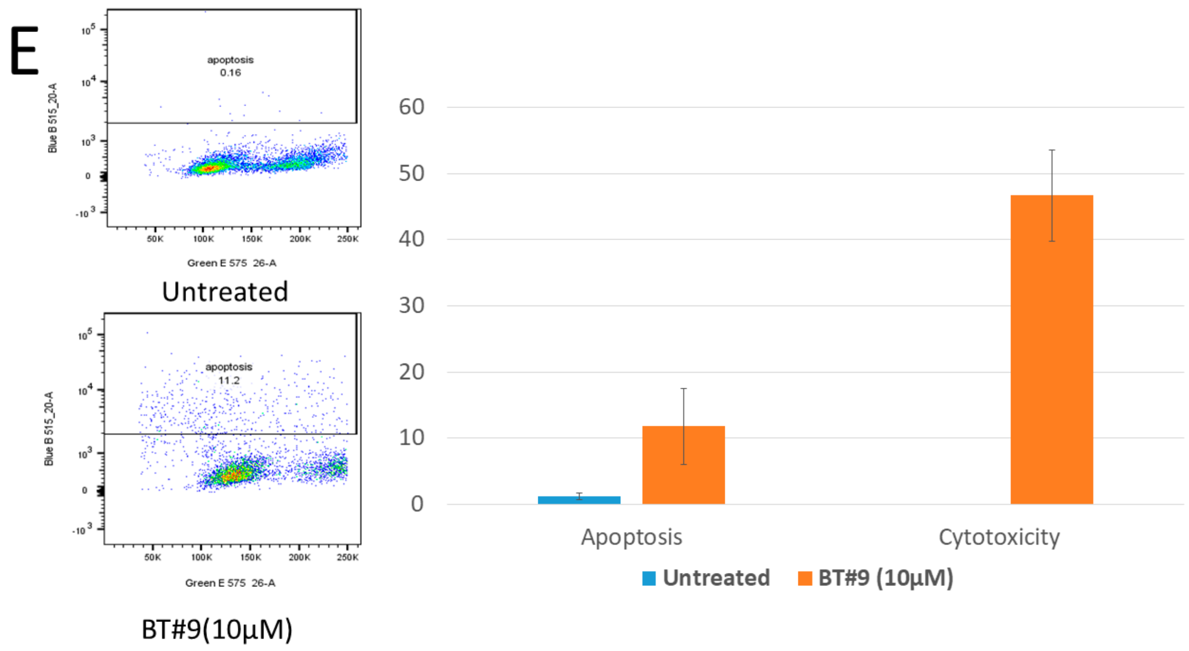

Supplemental Figure S2E (Magnified Figure 4E): TUNEL based Apo-BrdU apoptosis rate and MTS cell viability assay treated by BT#9.

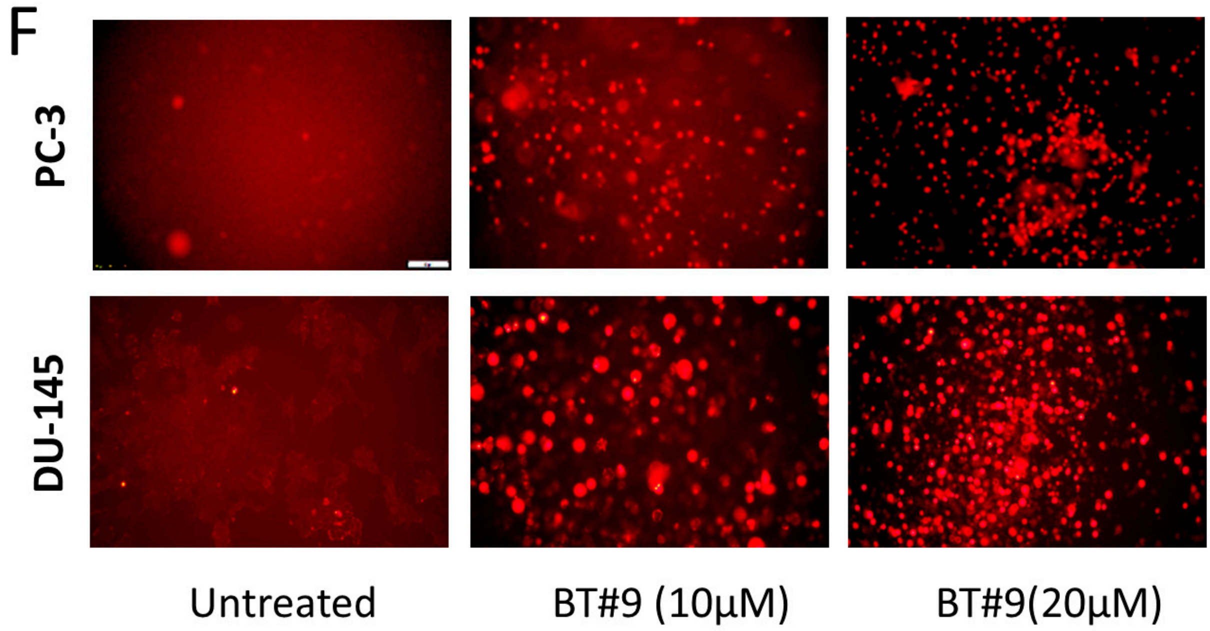

Supplemental Figure S2F (Magnified Figure 4F): PI staining by fluorescence microscopy; dramatic cell death treated by BT#9 at the concentration of 10 or 20 µM were detected.

G

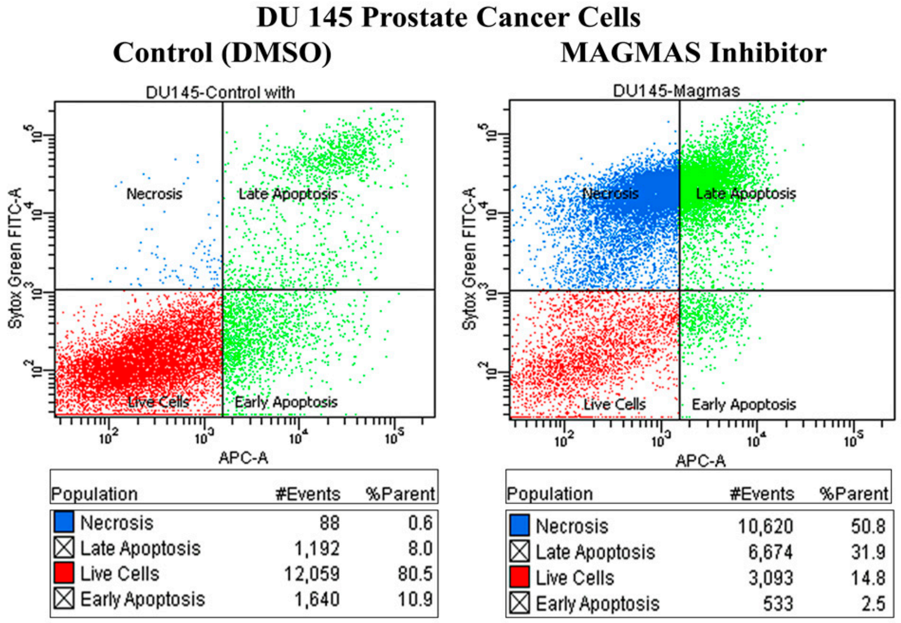

Population	#Events	%Parent
Necrosis	88	0.6
Late Apoptosis	1,192	8.0
Live Cells	12,059	80.5
Early Apoptosis	1,640	10.9

Population	#Events	%Parent
Necrosis	10,620	50.8
Late Apoptosis	6,674	31.9
Live Cells	3,093	14.8
Early Apoptosis	533	2.5

Supplemental Figure S2G (Magnified Figure 4G): Annexin V/SYTOX Green Assay for DU-145 cells treated by BT#9(10μM).

Supplementary Figure S3(Magnified Figure 5): The accumulation of ROS in Prostate cancer cells after BT#9 treatment.

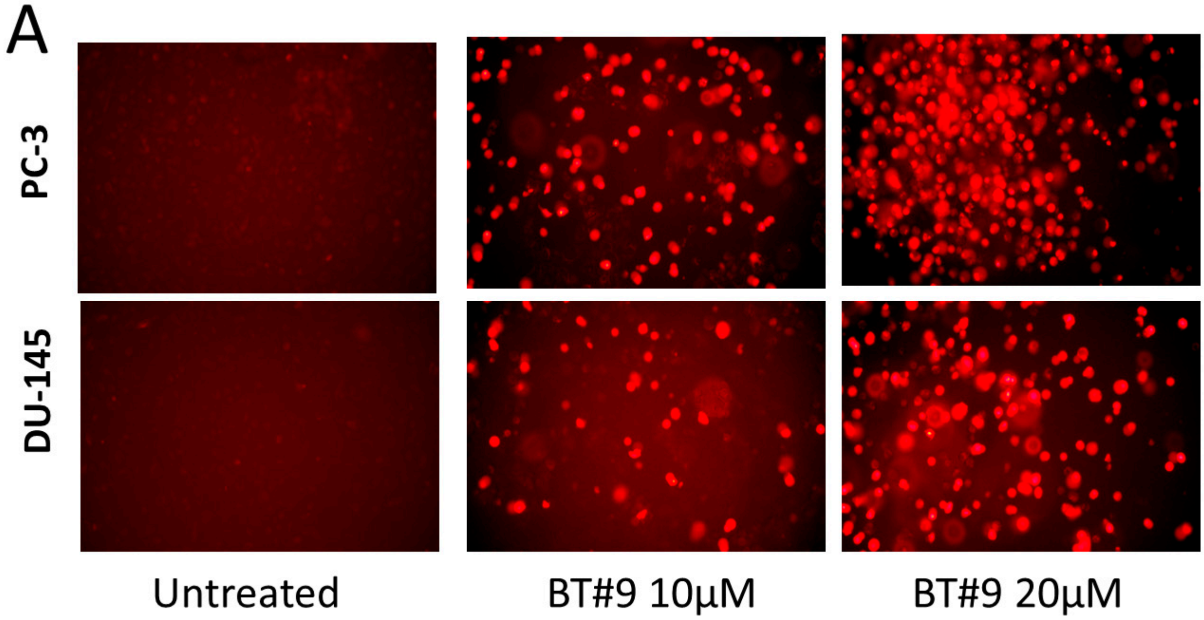

Supplemental Figure S3A (Magnified Figure 5A): Intra-mitochondria ROS of PC-3 and DU-145 cells detected by MitoSOX Red superoxide indicator after 10µM or 20µM BT#9 treatment.

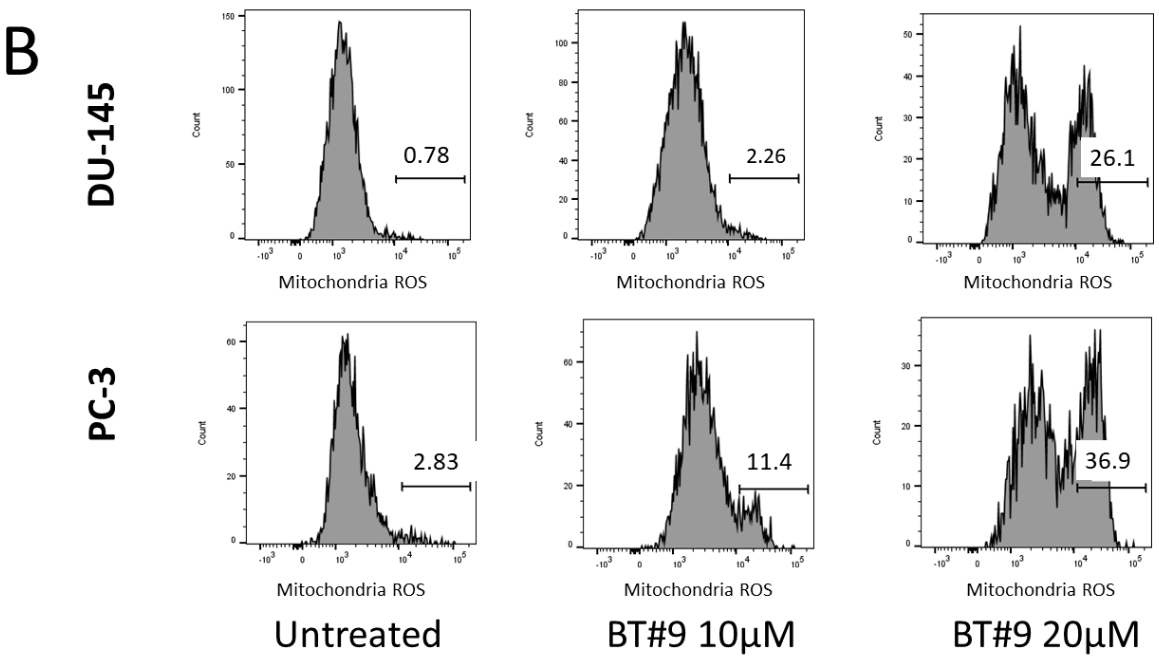

Supplemental Figure S3B (Magnified Figure 5B): Representative flow cytometry histograms of MitoSOX Red fluorescence (530 nm lasor, PE channel) in PC-3 and DU-145 cells treated by 10μM and 20μMBT#9. Percentage of MitoSox Red positive cells was analyzed and quantified by FlowJo Software.

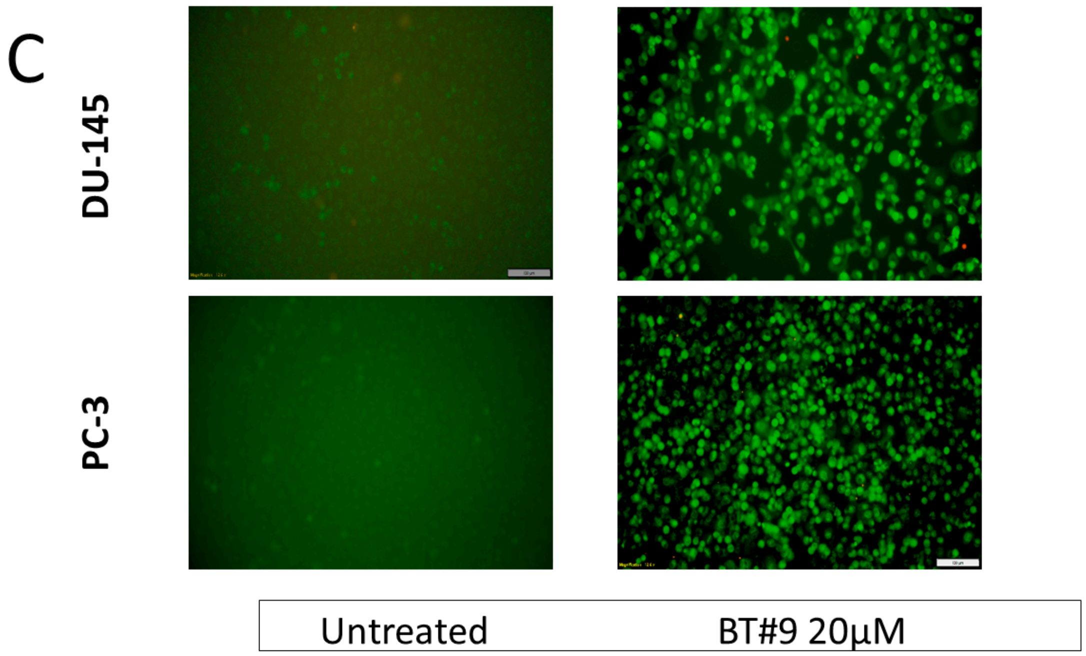

Supplemental Figure S3C (Magnified Figure 5C): PC-3 and DU-145 cells were treated by BT#9 for 24 h, stained by 5 μM CellROX Green Reagent, and Intracellular ROS was detected by fluorescence microcopy.

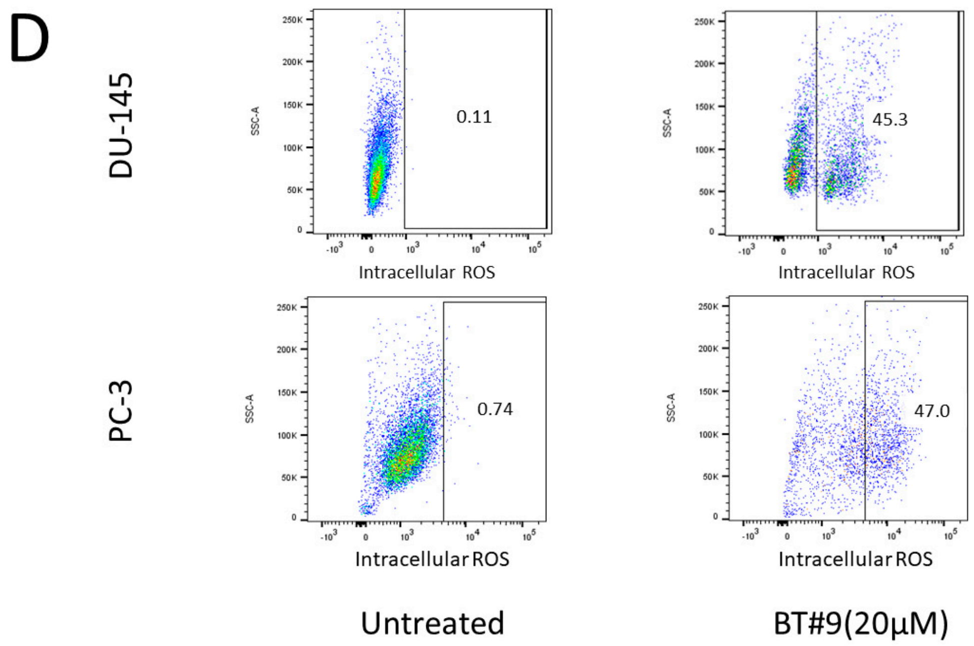

Supplemental Figure S3D (Magnified Figure 5D): FCM measured the intracellular ROS level in both PC-3 and DU-145 cells, showing significant ROS acculculation after BT#9 treatment (20 μM).

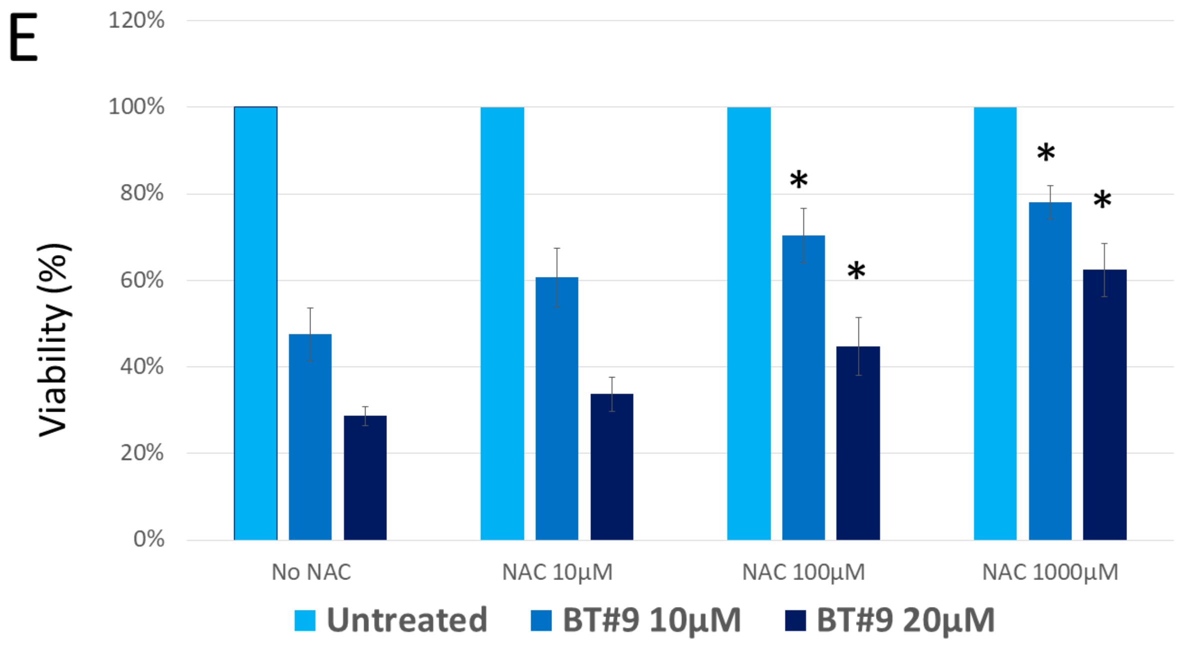

Supplemental Figure S3E (Magnified Figure 5E): NAC protected BT#9 induced cell death in a dose dependent way.

Supplementary Figure S4 (Magnified Figure 6): The combination of BT#9 and TRAIL or Docetaxel

A

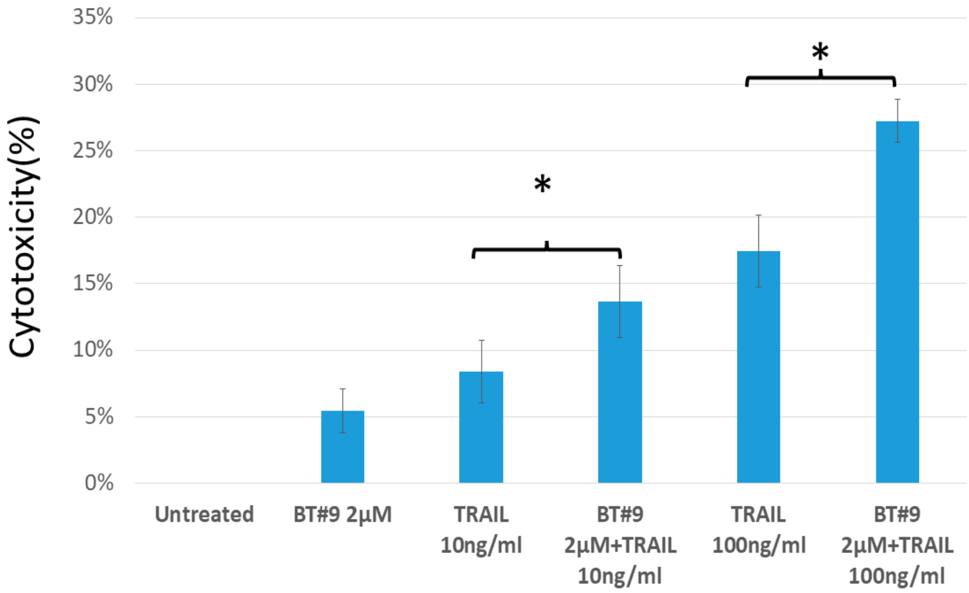

B

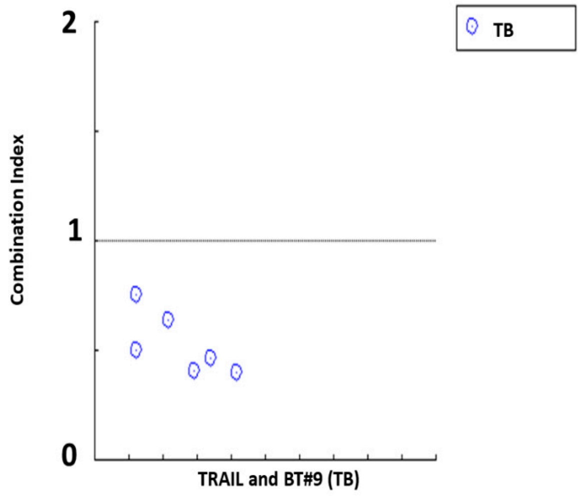

Supplemental Figure S4A and S4B (Magnified Figure 6A and 6B): The representative result of the combination of BT#9 (2μM) and TRAIL (10ng/ml or 100ng/ml). The whole CI of BT#9 (1,2 or 5μM) and TRAIL (10 or 100ng/ml) is between 0.22 and 0.72.

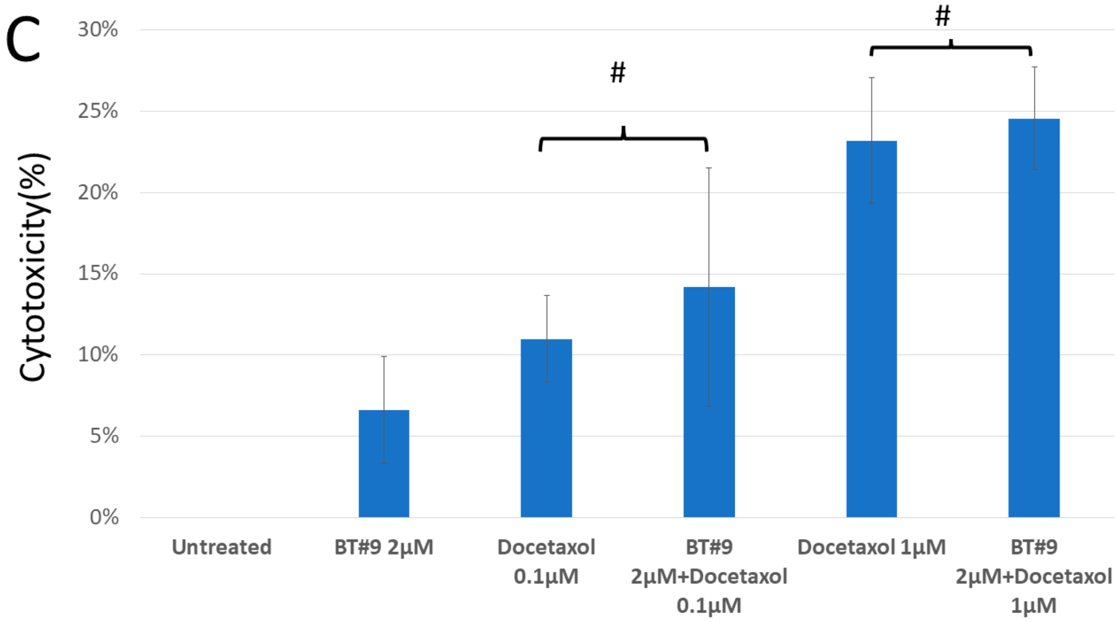

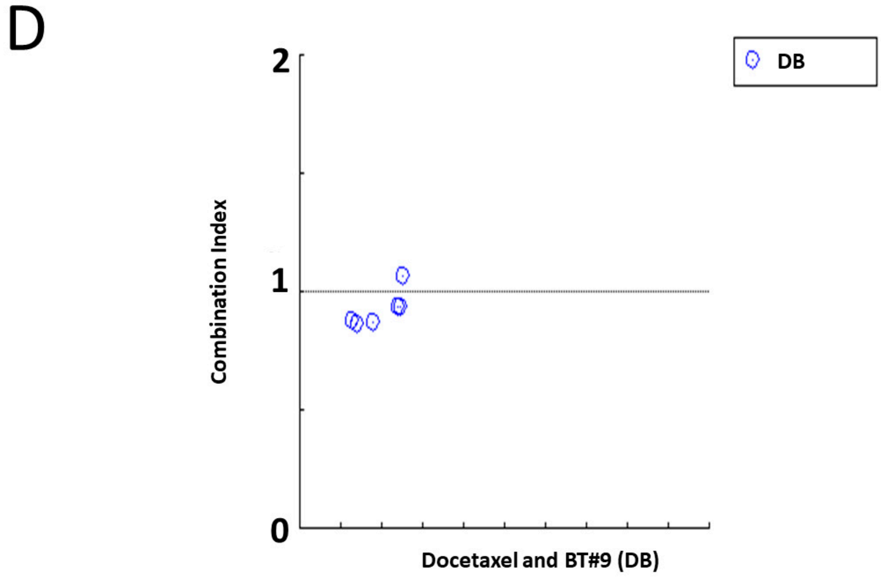

Supplemental Figure S4C and S4D (Magnified Figure 6C and 6D): The representative result of the combination between BT#9 (2μM) and Docetaxel (0.1μM or 1μM) The whole CI of the concurrent combination of BT#9 (1,2 or 5μM) and Docetaxel (0.1 or 1μM) is between 0.86 and 1.06.

E

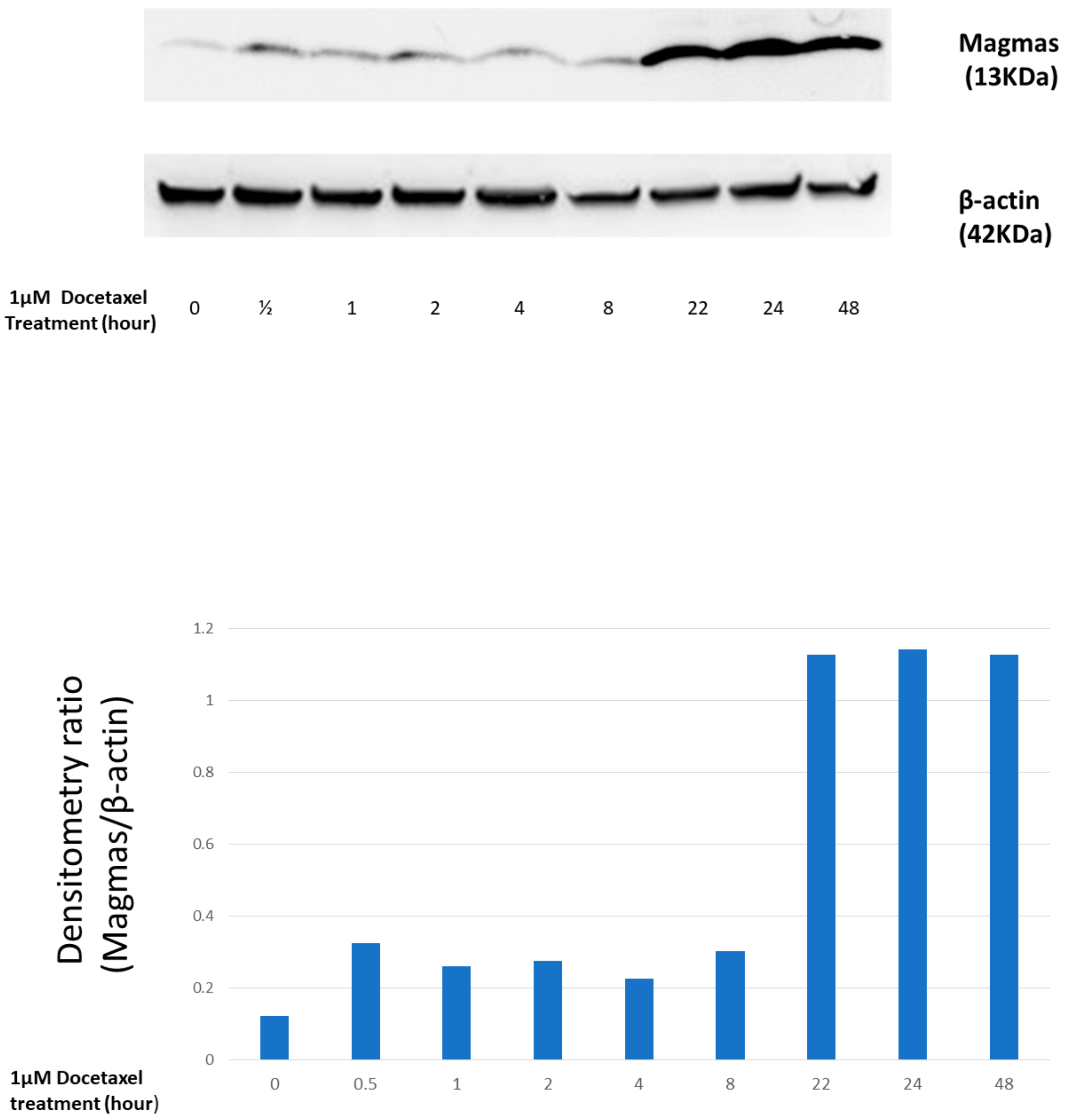
Magmas (13KDa)
β-actin (42KDa)
1μM Docetaxel Treatment (hour)
0
½
1
2
4
8
22
24
48
Densitometry ratio (Magmas/β-actin)
1.2
1
0.8
0.6
0.4
0.2
0
1μM Docetaxel treatment (hour)
0
0.5
1
2
4
8
22
24
48

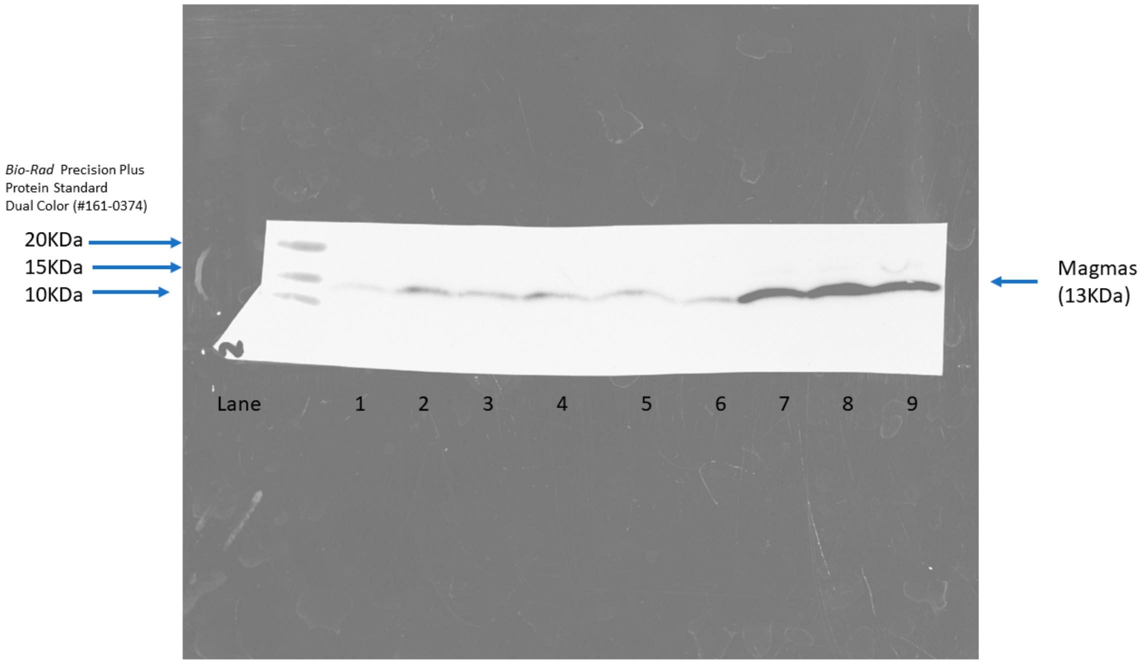

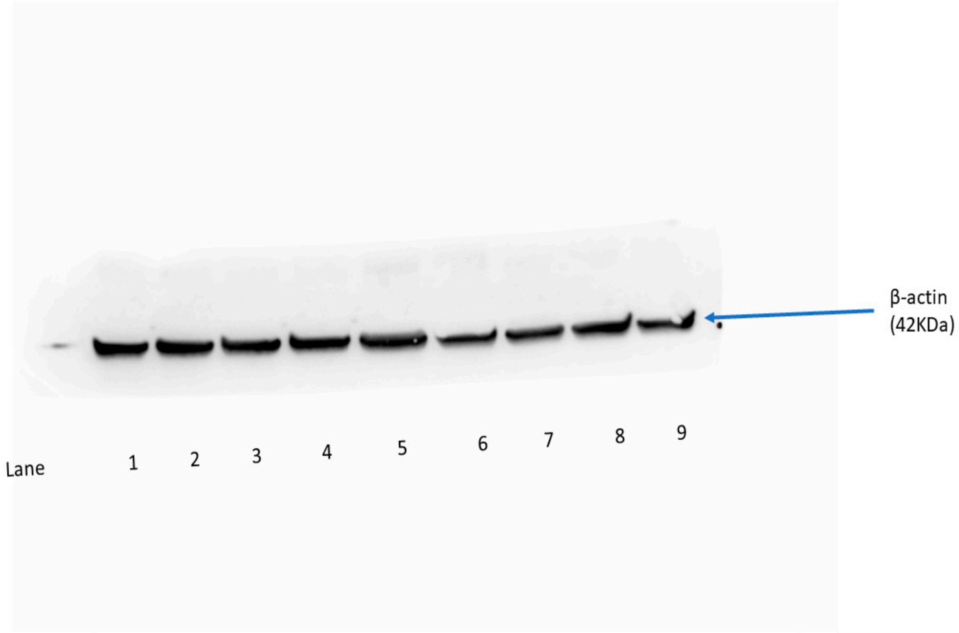

Supplemental Figure S4E (Magnified Figure 6E): The Magmas expression of PC-3 cells was upregulated around 24 hours or later after 1μM Docetaxel treatment.

F

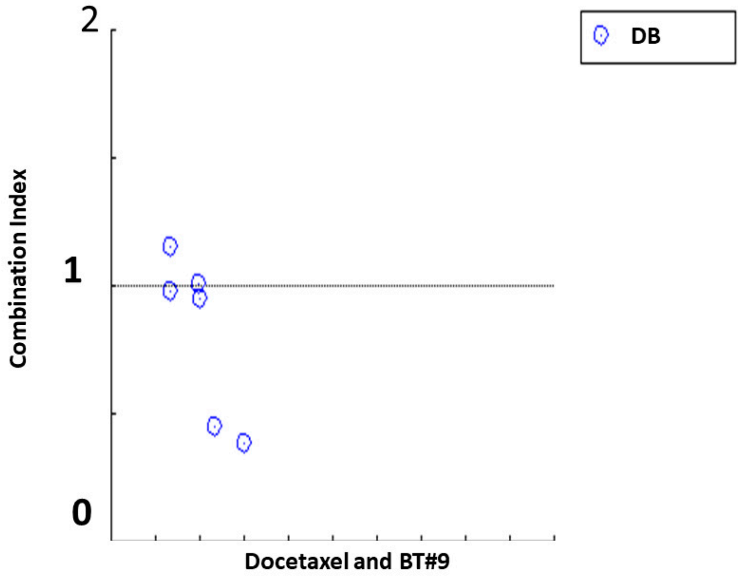

Supplemental Figure S4F (Magnified Figure 6F): The whole CI of the sequential combination of BT#9 (1,2 or 5μM) and Docetaxel (0.1 or 1μM) is between 0.39 and 1.15. Among all 6 dots, upper 4 dots (CI=0.95-1.15) represent CI for Docetaxel (0.1 or 1μM) and BT#9 (1 or 2 Mμ), lower 2 dots (CI=0.39-0.45) represent CI for Docetaxel (0.1 or 1μM) and BT#9(5μM).
